# Disturbances of intentionality in schizophrenia and in depression

**DOI:** 10.1192/j.eurpsy.2021.1994

**Published:** 2021-08-13

**Authors:** O. Dörr

**Affiliations:** Psychiatric And Mental Health, University of Chile, Santiago, Chile

**Keywords:** intentionality, phenomenology, schizophrenia, depression

## Abstract

**Introduction:**

IIntentionality constitutes an essential phenomenon of psychic life (Brentano, 1874; Husserl, 1917). Its disturbance can be expected in schizophrenia and depression.

**Objectives:**

To study how such fundamental psychic function is altered in these two diseases.

**Methods:**

Phenomenological method allows to deepen in the structure of complex phenomena. Husserl’s analysis of “consciousness of immanent time” helps in studying how intentionality functions in schizophrenia. In depression, we appeal to own previous phenomenological researches revealing three fundamental features: a specific change in body experience; inability to act, feel, think, etc. (inhibition); and alteration, inversion, and suspension of biorhythms.

**Results:**

The intentional arc connects the beginning and the end of a phrase. This arc will keep tenser, the bigger is the potency of the aim of my speech and my capacity to exclude inadequate associations. In schizophrenia intentional arc expands and appear “lax associations” (Bleuler, 1911) and “overinclusion” (Cameron, 1968). Fuchs (2005) argues that also the rest of schizophrenic symptoms represent disturbances of intentionality, e. g., in paranoid ideas an inversion occurs. In depression, its three essential phenomena can be interpreted as different forms of intentionality failure: the compromise of lived body and its consequent loss of transparence lead to incapacity of projecting oneself toward action and future. “Not being able to” (inhibition) means a detention of intentionality. Closely related appears the inability to anticipate. Finally, the alteration, inversion or suspension of biorhythms is temporal and insofar implicates a disturbance of intentionality.

**Conclusions:**

The main features of schizophrenia and depression represent specific forms of alteration of intentionality.

**Disclosure:**

No significant relationships.

